# Supplementary material for: Deletion of beta‐fructofuranosidase (invertase) genes is associated with sucrose content in Date Palm fruit
Source: Plant Direct. 2020 May 27;4(5):e00214. doi: 10.1002/pld3.214 (PMC7251787; doi:10.1002/pld3.214)
Supplement: Supplementary file 1 — Table S1 [file PLD3-4-e00214-s001.pdf]

Sample Code	Date Variety	Country of Pı	Eastern/Western Cultivar
01-ASL-AE	Aseela	Dubai, UAE	Eastern Cultivars
02-PRM1-IR	Piarom, Grac	Iran	Eastern Cultivars
04-RB-IR	Rabee	Iran	Eastern Cultivars
05-KHS-IR	Khasoy	Iran	Eastern Cultivars
06-YSH-AE	Ayash	UAE	Eastern Cultivars
07-MTZ-AE	Mumtaza	UAE	Eastern Cultivars
08-NWDR-AE	Nawader	UAE	Eastern Cultivars
09-ZML-KSA	Zamlee	KSA	Eastern Cultivars
10-SHSH-AE	Sheshe	UAE	Eastern Cultivars
11-MNF-AE	Menefi	UAE	Eastern Cultivars
12- BRN-AE	Berni	UAE	Eastern Cultivars
13-MBR-AE	Ambara	UAE	Eastern Cultivars
14-SLTN-AE	Sultana	UAE	Eastern Cultivars
15-JBR-AE	Jabri	UAE	Eastern Cultivars
16-BMN-AE	BuMaan	UAE	Eastern Cultivars
17-SFWN-AE	Safawin	UAE	Eastern Cultivars
18-SD-EG	Saeedi	Egypt	Western Cultivars
19-GG-EG	Gagee	Egypt	Western Cultivars
20-GHZL-EG	Ghazelee	Egypt	Western Cultivars
21-MLKB-EG	Malakabi	Egypt	Western Cultivars
22-BRM-IQ	Ibrahim (Ira	Iraq	Eastern Cultivars
23-MLMD-IQ	Um Alamod	Iraq	Eastern Cultivars
24-KHYR-IQ	Khiyara	Iraq	Eastern Cultivars
25-BRN-PK	Barani	Pakistan	Western Cultivars
26-SNGL-PK	Sangol	Pakistan	Eastern Cultivars
27-ASL-PK	Aseel	Pakistan	Eastern Cultivars
28-GRTH-PK	Gorakh	Pakistan	Western Cultivars
29-ZYK-PK	Zayaki	Pakistan	Western Cultivars
30-BRML-LY	Burmeel	Libya	Western Cultivars
31-BRN-LY	Berni	Libya	Western Cultivars
32-ABL-LY	Abel	Libya	Western Cultivars
34-SGDR-SA	Sagai dry	KSA	Eastern Cultivars
35-KHDR-SA	Khudry	KSA	Eastern Cultivars
36-AJWM-SA	Ajwa medina	KSA	Eastern Cultivars
37-SFWM-SA	Safawi - med	KSA	Eastern Cultivars
38-SGR-SA	Sagai - Rotal	KSA	Eastern Cultivars
39-RSHDY-SA	Rushydia	KSA	Eastern Cultivars
40-NBTST-SA	Naboot Sulta	KSA	Eastern Cultivars
41-SFR-SA	Sufri	KSA	Eastern Cultivars
42-SHSHR-SA	Shieshi Rotal	KSA	Eastern Cultivars
43-KHLS-QA	khlas	Qatar	Eastern Cultivars
44-MBRM-SA	Mabroom	KSA	Eastern Cultivars
45-MDJL-SA	Medjoul	KSA	Western Cultivars
46-NBTL-SA	Naboot Ali	KSA	Eastern Cultivars
47-AMBRM-SA	Amber - med	KSA	Eastern Cultivars
48-MBRM-QA	Mabroom	Qatar	Eastern Cultivars
49-SKR-QA	Sukary	Qatar	Eastern Cultivars

50-KHDR-QA	Khudry	Qatar	Eastern Cultivars
51-DRDT-QA	Dried dates	Qatar	Eastern Cultivars
52-SFR-QA	Sufri	Qatar	Eastern Cultivars
53-SFW-SA	Safawi	KSA	Eastern Cultivars
54-MBRM-SA	Mabroom	KSA	Eastern Cultivars
55-KHNZ-SA	Kheneizi	KSA	Eastern Cultivars
56-SQ-QA	Suqai	Qatar	Eastern Cultivars
57-SFW-SA	Safawi	KSA	Eastern Cultivars
58-AG-AE	Sagai	UAE	Eastern Cultivars
60-ALG-TN	Allig	Tunisia	Western Cultivars
62-GRNJ-TN	Grenja	Tunisia	Western Cultivars
64-DGTNR-TN	Deglet Nour	Tunisia	Western Cultivars
69-MDJL-US	Medjoul	California, U	Western Cultivars
70-SKR-SA	Sukkary	KSA	Eastern Cultivars
71-FGGHD-MA	Fegous-ghlid	Morocco	Western Cultivars
72-MJLA-MA	Majhool AA	Morocco	Western Cultivars
73-JHL-MA	Jihl	Morocco	Western Cultivars
74-TDMN-MA	Tadment	Morocco	Western Cultivars
75-SYR-MA	Sayer	Morocco	Western Cultivars
77-HFS-MA	Hafs	Morocco	Western Cultivars
78-BZGZ-MA	Bouzagza	Morocco	Western Cultivars
79-BKHD-MA	Boukhanda	Morocco	Western Cultivars
80-AGA-MA	Agalid	Morocco	Western Cultivars
81-LKL-MA	Lakhal	Morocco	Western Cultivars
82-NJD-MA	Najda	Morocco	Western Cultivars
83-WRDN-MA	Wahardan	Morocco	Western Cultivars
84-BDJJ-MA	Bid-djaj	Morocco	Western Cultivars
85-AZGHZ-MA	Azaghzaw	Morocco	Western Cultivars
86-BSLKH-MA	Bouslikh	Morocco	Western Cultivars
87-TZGRT-MA	Tazgaghzt	Morocco	Western Cultivars
88-TMRT-MA	Tamaright 2	Morocco	Western Cultivars
89-KLMR-MA	khalt-lhamm	Morocco	Western Cultivars
90-MJL-MA	Mejhool	Morocco	Western Cultivars
91-BLZT-MA	Belahzit	Morocco	Western Cultivars
92-SHTW-MA	Shetwia	Morocco	Western Cultivars
93-BSDN-MA	Booserdoon	Morocco	Western Cultivars
96-BKR-MA	Bekri-khder	Morocco	Western Cultivars
97-THMT-MA	Tahmoot 2	Morocco	Western Cultivars
98-BFGS-MA	Boufegous r	Morocco	Western Cultivars
99-MJN-MA	Maajoun	Morocco	Western Cultivars
100-BSKR-MA	Boussakri	Morocco	Western Cultivars
101-ADM-MA	Admoo	Morocco	Western Cultivars
103- TZW-MA	Tarzawa	Morocco	Western Cultivars
104-BZKR-MA	Boozekri	Morocco	Western Cultivars
105-ZGHL-EG	Zaghlool	Egypt	Western Cultivars
108-DGBD-DZ	Deglet Bayd	Algeria	Western Cultivars
109-DGNH-DZ	Deglet Nour	Algeria	Western Cultivars
112-ADAL-DZ	Adam adaal	Algeria	Western Cultivars

113-TBCHT-DZ	Tantebouchte	Algeria	Western Cultivars
114-FGS-DZ	Fegous	Algeria	Western Cultivars
117-DGNR-DZ	Deglet Nour	Algeria	Western Cultivars
119-TLMS-DZ	Telemsoua	Algeria	Western Cultivars
120-ADBJ-DZ	Adam bujam	Algeria	Western Cultivars
122-TMJT-DZ	Tamjhourt	Algeria	Western Cultivars
124-GHRS-DZ	Gharss	Algeria	Western Cultivars
126-TGBT-DZ	Tagarbushet	Algeria	Western Cultivars
128-DGLT-DZ	Deglet	Algeria	Western Cultivars
129-ADMN-DZ	Adam admaı	Algeria	Western Cultivars
131-HMR-DZ	Hameera	Algeria	Western Cultivars
133-HRY-DZ	Hamray	Algeria	Western Cultivars
136-ZHD-JO	Zehdi	Jordan	Eastern Cultivars
137-LULU-JO	Lulu	Jordan	Eastern Cultivars
139-SIWA-EG	Siwa	Egypt	Western Cultivars
142-KMSD-SD	Kulma soda	Sudan	Western Cultivars
143-GRDL-SD	Grundeela/C	Sudan	Western Cultivars
144-BRKW-SD	Barakawi	Sudan	Western Cultivars
145-STBN-SD	Sultana Bede	Sudan	Western Cultivars
146-ADQM-SA	Aydea Al Qas	KSA	Eastern Cultivars
147-KTR-AE	Qatarah/Kat	UAE	Eastern Cultivars
148-MSL-AE	Msalla	UAE	Eastern Cultivars
149-DBS-AE	Dabbas	UAE	Eastern Cultivars
150-SLG-AE	Sillege	UAE	Eastern Cultivars
151-HMSR-IR	Harmayi Sad	Iran	Eastern Cultivars
P3-ROE-US	Phoenix roeb	USA	*Phoenix* spp.
P8-CAN-US	Phoenix cana	USA	*Phoenix* spp.
P23-SYL-US	Phoenix sylve	USA	*Phoenix* spp.

Normalized Sucrose level (	PDK30s742521_60107b39		Normalized Coverage (at 42kb)
1.089172932	A/A	Hmz	1.24643098
0.878349282	A/A	Hmz	1.54767079
1.382392027	A/A	Hmz	1.32515484
1.765124555	A/A	Hmz	1.56997604
1.570051635	A/A	Hmz	1.61575605
1.475046211	A/A	Hmz	1.03459679
2.040314136	A/A	Hmz	1.41795733
17.9251462	A/A	Hmz	2.22176323
1.391777188	A/A	Hmz	0.89006993
1.449723757	A/A	Hmz	1.09313544
1.005702918	A/A	Hmz	1.39441918
1.481855956	A/A	Hmz	0.629399
0.774242424	A/A	Hmz	0.63959476
1.419252874	A/A	Hmz	0.58968589
2.038333333	A/A	Hmz	0.73172762
1.544528875	A/A	Hmz	0.66359245
1.196453901	A/A	Hmz	1.12741876
1.138589212	A/A	Hmz	1.69610021
17.63905683	A/A	Hmz	1.08041213
1.478601695	A/A	Hmz	1.32899949
1.329836512	A/A	Hmz	1.59440892
1.404736842	A/A	Hmz	1.35152504
0.953272251	A/A	Hmz	2.22907093
0.832510885	A/A	Hmz	1.66469862
1.753111111	A/A	Hmz	0.865877
1.654137447	A/A	Hmz	1.3521866
0.756491713	A/A	Hmz	1.39233547
1.375506757	A/T	Het	0.48208215
1.570978441	A/A	Hmz	1.27390931
1.126467066	A/A	Hmz	1.18488954
0.924346917	A/A	Hmz	1.62521258
1.199868074	A/A	Hmz	1.43602023
2.024708171	A/A	Hmz	1.12624842
1.756666667	A/A	Hmz	1.12900026
1.798561151	A/A	Hmz	0.50179269
1.281086519	A/A	Hmz	1.38136832
16.65119917	A/A	Hmz	1.15021517
1.436940299	A/A	Hmz	2.26509445
2.4086	A/A	Hmz	0.62770871
1.454929577	A/A	Hmz	1.36248563
1.790390879	A/A	Hmz	1.47368206
2.163362832	A/A	Hmz	1.32090117
1.616938519	A/T	Het	0.90049461
85.25272109	T/T	Del	0.05334187
1.065125241	A/A	Hmz	0.74013363
1.524882629	A/A	Hmz	0.92722421
71.72693333	T/T	Del	0.03659972

1.873472429	A/A	Hmz	0.97890036
0.818247647	A/A	Hmz	1.18311161
2.045411392	A/A	Hmz	0.62313859
1.49791411	A/A	Hmz	0.7996177
1.334446131	A/A	Hmz	0.963748
2.134061931	A/A	Hmz	0.95015655
1.843168317	A/A	Hmz	1.5236717
1.789959016	A/A	Hmz	0.65126995
1.264395887	A/A	Hmz	1.4330449
0.679461034	A/T	Het	0.63137758
0.472952381	A/T	Het	0.62833723
52.56560789	T/T	Del	0.0011957
1.428438662	A/A	Hmz	1.17959538
52.69944134	T/T	Del	0.04595855
0.730669546	A/A	Hmz	1.12001066
1.175206612	A/A	Hmz	0.88447212
4.378413737	A/A	Hmz	1.10583309
2.8134	A/A	Hmz	0.96548663
1.286157025	A/T	Het	0.38469433
1.154144621	A/T	Het	0.52727275
0.757045455	A/A	Hmz	1.34645869
2.04847512	A/A	Hmz	1.03348878
5.612742718	A/A	Hmz	1.21077842
0.429320988	A/A	Hmz	0.91817974
0.433650794	A/A	Hmz	1.27942266
1.264974619	A/A	Hmz	0.96248785
1.190458716	A/A	Hmz	0.98237478
1.044276094	A/T	Het	0.88590003
0.80020284	A/A	Hmz	0.82895199
1.39960733	A/T	Het	0.51720624
0.742933333	A/A	Hmz	1.25219595
0.923933649	A/A	Hmz	0.85357205
1.141397849	A/T	Het	1.11190601
0.59245614	A/A	Hmz	1.08471815
0.611807229	A/T	Het	0.77788523
3.222775801	A/A	Hmz	1.24606318
1.737268519	A/A	Hmz	1.19702186
3.591553134	A/A	Hmz	1.17897871
0.872565321	A/A	Hmz	1.35011364
2.874239351	A/A	Hmz	1.34541843
31.10215463	A/A	Hmz	0.55144508
3.566889632	A/A	Hmz	1.24532801
0.774262735	A/A	Hmz	1.30993877
0.820679012	A/T	Het	0.81184461
1.008787879	A/A	Hmz	1.09582132
26.27088353	A/T	Het	0.59119536
112.8505443	T/T	Del	0.05400346
1.703168317	A/A	Hmz	1.49884747

	1.338095238	A/A	Hmz	1.68890185
	1.31322314	A/A	Hmz	0.87862796
	75.20625698	T/T	Del	0.0059497
	1.81677116	A/A	Hmz	1.4611789
	1.532228916	A/A	Hmz	2.42476358
	0.97997416	A/A	Hmz	1.07632499
	2.923976608	A/T	Het	0.73070717
	0.959923175	A/A	Hmz	1.05029995
	1.199356223	A/A	Hmz	0.7657382
	1.209561753	A/A	Hmz	1.62262182
	1.430617284	A/A	Hmz	1.3434841
	0.878705281	A/A	Hmz	1.63064879
	1.050362319	A/A	Hmz	0.87971127
	1.445110821	A/A	Hmz	1.97064892
	1.949621212	A/A	Hmz	1.06887645
	2.727098675	A/T	Het	1.09850015
	1.558278146	A/A	Hmz	1.39979923
	2.370978441	A/A	Hmz	1.19026515
	2.234022258	A/A	Hmz	0.61495576
	7.286829268	A/T	Het	0.59796164
	3.089803922	A/A	Hmz	0.64799223
	1.557589286	A/A	Hmz	1.11123394
	1.817891374	A/A	Hmz	1.72081542
	1.014035088	A/A	Hmz	1.39594546
	1.881322957	A/A	Hmz	1.15843749
N/A		A/A	Hmz	N/A
N/A		A/A	Hmz	N/A
N/A		A/A	Hmz	N/A
