# Supplementary material for: Deletion of beta‐fructofuranosidase (invertase) genes is associated with sucrose content in Date Palm fruit
Source: Plant Direct. 2020 May 27;4(5):e00214. doi: 10.1002/pld3.214 (PMC7251787; doi:10.1002/pld3.214)
Supplement: Supplementary file 2 — Table S2 [file PLD3-4-e00214-s002.pdf]

CHR	SNP	Unadjusted P-value
PDK30s1089221_151057	PDK30s1089221s3650	6.70E-21
PDK30s1089221_151057	PDK30s1089221s8086	3.92E-23
PDK30s1089221_151057	PDK30s1089221s22734	2.77E-23
PDK30s1089221_151057	PDK30s1089221s41323	2.50E-24
PDK30s1089221_151057	PDK30s1089221s46879	1.45E-23
PDK30s1089221_151057	PDK30s1089221s66062	1.26E-20
PDK30s1089221_151057	PDK30s1089221s69340	6.80E-21
PDK30s1089221_151057	PDK30s1089221s97882	3.28E-25
PDK30s1089221_151057	PDK30s1089221s124053	1.51E-23
PDK30s6550997_92357	PDK30s6550997s7860	3.87E-23
PDK30s6550997_92357	PDK30s6550997s9214	1.20E-24
PDK30s6550997_92357	PDK30s6550997s13026	3.31E-24
PDK30s6550997_92357	PDK30s6550997s15803	5.67E-17
PDK30s6550997_92357	PDK30s6550997s16819	3.87E-23
PDK30s6550997_92357	PDK30s6550997s18172	7.81E-22
PDK30s6550997_92357	PDK30s6550997s21249	3.87E-23
PDK30s6550997_92357	PDK30s6550997s27268	9.58E-21
PDK30s6550997_92357	PDK30s6550997s28841	4.57E-23
PDK30s6550997_92357	PDK30s6550997s29317	5.80E-25
PDK30s6550997_92357	PDK30s6550997s32095	6.39E-23
PDK30s6550997_92357	PDK30s6550997s36012	6.39E-23
PDK30s673261_40461	PDK30s673261s19081	2.25E-21
PDK30s673261_40461	PDK30s673261s20069	1.60E-19
PDK30s673261_40461	PDK30s673261s31257	6.68E-17
PDK30s673261_40461	PDK30s673261s31526	5.40E-19
PDK30s684281_65292	PDK30s684281s34548	4.54E-18
PDK30s684281_65292	PDK30s684281s61741	4.54E-18
PDK30s688281_25741	PDK30s688281s5529	3.21E-25
PDK30s712321_42322	PDK30s712321s2106	1.61E-17
PDK30s712321_42322	PDK30s712321s7577	4.91E-17
PDK30s712321_42322	PDK30s712321s9595	3.92E-18
PDK30s712321_42322	PDK30s712321s9611	1.62E-17
PDK30s712321_42322	PDK30s712321s14167	1.82E-18
PDK30s712321_42322	PDK30s712321s16464	2.42E-18
PDK30s712321_42322	PDK30s712321s23271	1.25E-20
PDK30s712321_42322	PDK30s712321s37300	3.82E-20
PDK30s712321_42322	PDK30s712321s40050	1.33E-17
PDK30s727751_44958	PDK30s727751s1317	3.87E-23
PDK30s739461_75594	PDK30s739461s32268	6.01E-21
PDK30s739461_75594	PDK30s739461s35132	7.77E-21
PDK30s739461_75594	PDK30s739461s45779	4.43E-17
PDK30s739461_75594	PDK30s739461s47359	2.52E-19
PDK30s742521_60107	PDK30s742521s39533	6.13E-18
PDK30s742521_60107	PDK30s742521s40462	4.04E-17
PDK30s909171_124968	PDK30s909171s719	1.35E-18
PDK30s909171_124968	PDK30s909171s51663	2.37E-21
PDK30s909171_124968	PDK30s909171s52984	1.61E-18

PDK30s909171_124968	PDK30s909171s54027	5.99E-22
PDK30s909171_124968	PDK30s909171s54042	4.69E-18
PDK30s909171_124968	PDK30s909171s54048	7.00E-25
PDK30s909171_124968	PDK30s909171s54188	2.19E-17
PDK30s909171_124968	PDK30s909171s54291	1.85E-17
PDK30s909171_124968	PDK30s909171s54312	4.26E-19
PDK30s909171_124968	PDK30s909171s54333	8.88E-25
PDK30s909171_124968	PDK30s909171s54391	1.14E-18
PDK30s909171_124968	PDK30s909171s54491	3.27E-19
PDK30s909171_124968	PDK30s909171s54521	1.16E-18
PDK30s909171_124968	PDK30s909171s54586	1.40E-22
PDK30s909171_124968	PDK30s909171s54624	7.00E-18
PDK30s909171_124968	PDK30s909171s56183	5.23E-17
PDK30s909171_124968	PDK30s909171s57072	2.19E-19
PDK30s909171_124968	PDK30s909171s67990	1.11E-21
PDK30s909171_124968	PDK30s909171s74408	9.06E-23
PDK30s909171_124968	PDK30s909171s75037	3.45E-20
PDK30s909171_124968	PDK30s909171s75173	4.05E-19
PDK30s909171_124968	PDK30s909171s75308	2.97E-22
PDK30s909171_124968	PDK30s909171s79171	2.58E-17
PDK30s909171_124968	PDK30s909171s87969	2.58E-17

GC	QQ	BONFERRON	HOLM	SIDAK_SS	SIDAK_SD	FDR_BH
4.55E-14	1.79E-05	2.16E-14	2.16E-14	2.15E-14	2.15E-14	3.71E-16
1.13E-15	1.01E-05	1.26E-16	1.26E-16	1.11E-16	1.11E-16	3.82E-18
8.09E-16	8.55E-06	8.91E-17	8.91E-17	1.11E-16	1.11E-16	3.18E-18
1.29E-16	5.75E-06	8.03E-18	8.03E-18	INF	INF	4.01E-19
4.72E-16	7.62E-06	4.67E-17	4.67E-17	INF	INF	1.87E-18
7.84E-14	1.94E-05	4.05E-14	4.05E-14	4.05E-14	4.05E-14	6.43E-16
4.92E-14	1.82E-05	2.19E-14	2.19E-14	2.19E-14	2.19E-14	3.71E-16
2.71E-17	2.33E-06	1.06E-18	1.06E-18	INF	INF	1.30E-19
5.10E-16	7.93E-06	4.85E-17	4.85E-17	INF	INF	1.87E-18
1.04E-15	8.86E-06	1.24E-16	1.24E-16	1.11E-16	1.11E-16	3.82E-18
7.36E-17	5.44E-06	3.85E-18	3.85E-18	INF	INF	2.14E-19
1.61E-16	6.37E-06	1.07E-17	1.07E-17	INF	INF	5.07E-19
3.21E-11	3.75E-05	1.82E-10	1.82E-10	1.82E-10	1.82E-10	1.51E-12
1.04E-15	9.17E-06	1.24E-16	1.24E-16	1.11E-16	1.11E-16	3.82E-18
9.97E-15	1.51E-05	2.51E-15	2.51E-15	2.55E-15	2.55E-15	5.13E-17
1.04E-15	9.48E-06	1.24E-16	1.24E-16	1.11E-16	1.11E-16	3.82E-18
5.49E-14	1.88E-05	3.08E-14	3.08E-14	3.09E-14	3.09E-14	5.05E-16
1.15E-15	1.07E-05	1.47E-16	1.47E-16	1.11E-16	1.11E-16	4.20E-18
4.21E-17	4.20E-06	1.87E-18	1.87E-18	INF	INF	1.33E-19
1.78E-15	1.10E-05	2.06E-16	2.06E-16	2.22E-16	2.22E-16	5.55E-18
1.52E-15	1.13E-05	2.06E-16	2.06E-16	2.22E-16	2.22E-16	5.55E-18
2.19E-14	1.60E-05	7.23E-15	7.23E-15	7.22E-15	7.22E-15	1.39E-16
5.04E-13	2.19E-05	5.13E-13	5.13E-13	5.13E-13	5.13E-13	7.23E-15
3.82E-11	3.90E-05	2.15E-10	2.15E-10	2.15E-10	2.15E-10	1.71E-12
1.17E-12	2.47E-05	1.74E-12	1.74E-12	1.74E-12	1.74E-12	2.17E-14
5.65E-12	2.94E-05	1.46E-11	1.46E-11	1.46E-11	1.46E-11	1.52E-13
5.65E-12	2.97E-05	1.46E-11	1.46E-11	1.46E-11	1.46E-11	1.52E-13
2.67E-17	1.40E-06	1.03E-18	1.03E-18	INF	INF	1.30E-19
1.67E-11	3.22E-05	5.17E-11	5.17E-11	5.17E-11	5.17E-11	4.96E-13
3.07E-11	3.68E-05	1.58E-10	1.58E-10	1.58E-10	1.58E-10	1.33E-12
5.09E-12	2.91E-05	1.26E-11	1.26E-11	1.26E-11	1.26E-11	1.34E-13
1.39E-11	3.25E-05	5.21E-11	5.21E-11	5.21E-11	5.21E-11	4.96E-13
2.93E-12	2.75E-05	5.85E-12	5.85E-12	5.85E-12	5.85E-12	6.58E-14
3.60E-12	2.81E-05	7.78E-12	7.78E-12	7.78E-12	7.78E-12	8.55E-14
7.80E-14	1.91E-05	4.02E-14	4.02E-14	4.02E-14	4.02E-14	6.43E-16
1.77E-13	2.04E-05	1.23E-13	1.23E-13	1.23E-13	1.23E-13	1.86E-15
1.22E-11	3.19E-05	4.28E-11	4.28E-11	4.28E-11	4.28E-11	4.15E-13
1.04E-15	9.79E-06	1.24E-16	1.24E-16	1.11E-16	1.11E-16	3.82E-18
5.29E-14	1.76E-05	1.93E-14	1.93E-14	1.93E-14	1.93E-14	3.39E-16
4.98E-14	1.85E-05	2.50E-14	2.50E-14	2.50E-14	2.50E-14	4.17E-16
2.86E-11	3.65E-05	1.43E-10	1.43E-10	1.43E-10	1.43E-10	1.21E-12
7.03E-13	2.28E-05	8.11E-13	8.11E-13	8.11E-13	8.11E-13	1.10E-14
7.00E-12	3.03E-05	1.97E-11	1.97E-11	1.97E-11	1.97E-11	2.01E-13
2.68E-11	3.59E-05	1.30E-10	1.30E-10	1.30E-10	1.30E-10	1.12E-12
2.12E-12	2.63E-05	4.34E-12	4.34E-12	4.34E-12	4.34E-12	5.10E-14
2.28E-14	1.63E-05	7.63E-15	7.63E-15	7.66E-15	7.66E-15	1.44E-16
2.61E-12	2.72E-05	5.17E-12	5.17E-12	5.17E-12	5.17E-12	5.88E-14

8.19E-15	1.45E-05	1.93E-15	1.93E-15	1.89E-15	1.89E-15	4.10E-17
5.78E-12	3.00E-05	1.51E-11	1.51E-11	1.51E-11	1.51E-11	1.56E-13
4.87E-17	4.51E-06	2.25E-18	2.25E-18	INF	INF	1.47E-19
1.66E-11	3.34E-05	7.04E-11	7.04E-11	7.04E-11	7.04E-11	6.51E-13
1.49E-11	3.31E-05	5.96E-11	5.96E-11	5.96E-11	5.96E-11	5.57E-13
1.03E-12	2.41E-05	1.37E-12	1.37E-12	1.37E-12	1.37E-12	1.76E-14
5.85E-17	5.13E-06	2.86E-18	2.86E-18	INF	INF	1.68E-19
1.31E-12	2.53E-05	3.68E-12	3.68E-12	3.68E-12	3.68E-12	4.48E-14
8.49E-13	2.32E-05	1.05E-12	1.05E-12	1.05E-12	1.05E-12	1.40E-14
1.85E-12	2.56E-05	3.73E-12	3.73E-12	3.73E-12	3.73E-12	4.49E-14
2.76E-15	1.38E-05	4.51E-16	4.51E-16	4.44E-16	4.44E-16	1.00E-17
7.61E-12	3.09E-05	2.25E-11	2.25E-11	2.25E-11	2.25E-11	2.25E-13
3.32E-11	3.71E-05	1.68E-10	1.68E-10	1.68E-10	1.68E-10	1.40E-12
5.99E-13	2.25E-05	7.06E-13	7.06E-13	7.06E-13	7.06E-13	9.67E-15
1.29E-14	1.57E-05	3.56E-15	3.56E-15	3.55E-15	3.55E-15	6.97E-17
1.52E-15	1.17E-05	2.92E-16	2.92E-16	3.33E-16	3.33E-16	7.67E-18
1.65E-13	1.97E-05	1.11E-13	1.11E-13	1.11E-13	1.11E-13	1.73E-15
9.91E-13	2.35E-05	1.30E-12	1.30E-12	1.30E-12	1.30E-12	1.71E-14
4.84E-15	1.41E-05	9.56E-16	9.56E-16	9.99E-16	9.99E-16	2.08E-17
1.73E-11	3.40E-05	8.31E-11	8.31E-11	8.31E-11	8.31E-11	7.49E-13
1.95E-11	3.43E-05	8.31E-11	8.31E-11	8.31E-11	8.31E-11	7.49E-13

FDR_BY
5.77E-15
5.95E-17
4.95E-17
6.25E-18
2.90E-17
1.00E-14
5.77E-15
2.02E-18
2.90E-17
5.95E-17
3.33E-18
7.90E-18
2.35E-11
5.95E-17
7.98E-16
5.95E-17
7.86E-15
6.53E-17
2.07E-18
8.64E-17
8.64E-17
2.16E-15
1.13E-13
2.65E-11
3.38E-13
2.37E-12
2.37E-12
2.02E-18
7.72E-12
2.06E-11
2.09E-12
7.72E-12
1.02E-12
1.33E-12
1.00E-14
2.90E-14
6.46E-12
5.95E-17
5.28E-15
6.48E-15
1.88E-11
1.71E-13
3.13E-12
1.75E-11
7.94E-13
2.24E-15
9.15E-13

6.38E-16
2.42E-12
2.29E-18
1.01E-11
8.66E-12
2.73E-13
2.61E-18
6.97E-13
2.18E-13
6.99E-13
1.56E-16
3.51E-12
2.18E-11
1.51E-13
1.09E-15
1.19E-16
2.70E-14
2.65E-13
3.24E-16
1.17E-11
1.17E-11
