# Supplementary material for: Deletion of beta‐fructofuranosidase (invertase) genes is associated with sucrose content in Date Palm fruit
Source: Plant Direct. 2020 May 27;4(5):e00214. doi: 10.1002/pld3.214 (PMC7251787; doi:10.1002/pld3.214)
Supplement: Supplementary file 3 — Table S3 [file PLD3-4-e00214-s003.pdf]

Gene Name	Description	Length
PDK30s673261g001	transcription factor e2f	377
PDK30s673261g002	protein	195
PDK30s673261g003	pathogenicity protein path531-like protein	261
PDK30s673261g004	protein	202
PDK30s688281g001	40s ribosomal protein s25-1	229
PDK30s688281g002	PREDICTED: hypothetical protein [Vitis vinifera]	241
PDK30s688281g003	rac gtpase activating protein 1	468
PDK30s712321g001	nhl1	184
PDK30s712321g002	protein	594
PDK30s909171g001	---NA---	148
PDK30s909171g002	protein	269
PDK30s909171g003	protein	285
PDK30s909171g004	protein	987
PDK30s909171g005	membrane-associated phospholipid phosphatase	238
PDK30s909171g006	basic leucine zipper protein	519
PDK30s909171g007	cell division protein	198
PDK30s909171g008	plastid division protein	373
PDK30s909171g009	protein	145
PDK30s909171g010	glucan endo- -beta-glucosidase	388
PDK30s909171g011	protein	393
PDK30s909171g012	copia-type polyprotein	557
PDK30s909171g013	nbs-lrr resistance protein	405
PDK30s909171g014	soul heme-binding protein	277
PDK30s909171g015	nbs-lrr resistance protein	888
PDK30s742521g001	protein	403
PDK30s742521g002	chaperone protein dnaj-related-like	293
PDK30s742521g003	40s ribosomal protein s14	150
PDK30s742521g004	ubiquitin fusion protein	128
PDK30s742521g005	cell wall invertase	567
PDK30s1089221g001	n-acetyltransferase esco1	383
PDK30s1089221g002	glycosyltransferase family 4 protein	411
PDK30s1089221g003	protein	340
PDK30s1089221g004	ribosomal protein s8e	141
PDK30s1089221g005	digalactosyldiacylglycerol synthase 1	815
PDK30s1089221g006	almt1	451
PDK30s1089221g007	lhw (lonesome highway) protein homodimerization t	162
PDK30s1089221g008	helix-loop-helix dna-binding	650
PDK30s1089221g009	protein	673
PDK30s1089221g010	growth inhibition and differentiation-related protein	261
PDK30s1089221g011	growth inhibition and differentiation-related protein	147
PDK30s1089221g012	protein	404
PDK30s1089221g013	protein	268
PDK30s1089221g014	protein	1051
PDK30s1089221g015	f-box family protein	329
PDK30s1089221g016	set domain protein	370
PDK30s739461g001	cationic amino acid	553
PDK30s739461g002	protein	582

PDK30s739461g003	rav-like b3 domain dna binding protein	209
PDK30s739461g004	---NA---	130
PDK30s739461g005	lysosomal pro-x	283
PDK30s739461g006	pentatricopeptide repeat-containing	347
PDK30s739461g007	b-type cyclin	178
PDK30s739461g008	splicing arginine serine-	546
PDK30s739461g009	ubiquitin-conjugating enzyme family protein	137
PDK30s727751g001	protein	703
PDK30s727751g002	abc transporter related	638
PDK30s727751g003	protein	743
PDK30s727751g004	small nuclear ribonucleoprotein d2	99
PDK30s727751g005	ifa binding protein	865
PDK30s6550997g001	at5g03870	237
PDK30s6550997g002	nectarin iii	568
PDK30s6550997g003	retrotransposon unclassified	170
PDK30s6550997g004	protein	595
PDK30s6550997g005	rna binding	709
PDK30s6550997g006	histone h3	130
PDK30s6550997g007	---NA---	139
PDK30s6550997g008	retrotransposon ty3-gypsy subclass	146
PDK30s6550997g009	retrotransposon ty3-gypsy sub-class	289
PDK30s6550997g010	nb-arc domain containing expressed	144
PDK30s684281g001	2-cys peroxiredoxin-like protein	177
PDK30s684281g002	dna binding	1373
PDK30s684281g003	protein	307
PDK30s684281g004	heat shock -like	787
PDK30s684281g005	5 -3 exoribonuclease 2	713

P-value	% similarity	Gene Ontology Information		
9.59E-135	79.25%	C:nucleoplasm; P:cell cycle		IPR003316; IPR011991
1.27E-41	73.55%	C:membrane; C:plastid; C:		IPR009688; SignalP (SIGNALP)
5.79E-54	71.35%	C:cytoplasm		IPR004345
1.25E-33	68.35%	C:intracellular; C:cytoplasm		IPR006458
1.38E-11	99.00%	C:ribosome; C:plastid		IPR000897; IPR004977; IPR007648
1.74E-36	54.62%			IPR021410
2.55E-156	73.10%	C:plastid		IPR000095; IPR000198
3.78E-40	79.80%	P:response to stress; P:res		IPR004864; SignalP (SIGNALP)
0	85.20%	F:protein binding; P:transp		IPR004140
				no IPS match
1.08E-35	86.30%	C:plastid; P:response to st		no IPS match
9.26E-57	64.50%	F:RNA binding; P:RNA-dep		no IPS match
2.29E-160	57.50%	F:molecular_function; P:bi		no IPS match
2.12E-86	75.70%	F:kinase activity; P:protein		IPR000340; G3DSA:3.90.190.10 (GENE3
2.98E-123	70.70%	F:transcription factor activ		IPR004827; IPR011616
7.62E-40	95.55%	C:cellular_co	EC:3.6.5.1; E	IPR000158; IPR003008
1.30E-77	88.20%	F:protein bin	EC:3.6.5.1; E	IPR000158; IPR003008; IPR018316; PF1
8.19E-25	75.20%	F:molecular_function; P:bi		no IPS match
4.80E-148	82.20%	F:hydrolase a	EC:3.2.1.0	IPR000490; IPR013781
1.72E-131	80.95%	C:membrane; C:plastid		no IPS match
7.34E-74	72.75%	F:binding; P:cellular proces		IPR013103
8.94E-100	63.10%	F:ATP binding; P:apoptosis		IPR000767; IPR002182
3.26E-104	77.60%	C:membrane; C:thylakoid;		IPR006917
4.25E-154	55.25%	F:ATP binding; P:apoptosis		IPR000767; IPR002182
7.69E-113	63.95%	C:mitochondrion		IPR021099
1.35E-116	83.25%	P:protein metabolic proces		no IPS match
4.42E-66	99.35%	C:ribosome;	EC:3.6.5.3	IPR001971
2.80E-69	98.10%	P:translation	EC:3.6.5.3	IPR000626; IPR001975; IPR019956; G3D
0	74.90%	F:hydrolase a	EC:3.2.1.0	IPR001362; IPR013148; IPR013189; Sigr
5.59E-81	66.30%	F:transferase activity		no IPS match
3.47E-145	69.45%	P:biosynthetic process; C:p		IPR001296
2.72E-66	62.60%	F:transcription regulator a		IPR004827
2.39E-24	49.00%	F:structural constituent of		IPR022309
0	82.50%	C:mitochond	EC:2.4.1.241;	IPR001296
1.97E-148	76.40%	C:membrane		IPR020966
1.76E-37	60.80%	P:multicellular organismal		SignalP (SIGNALP)
1.76E-127	64.70%	F:transcription regulator a		IPR001092; SignalP (SIGNALP)
4.06E-169	62.00%	F:DNA binding		IPR001356; IPR006563; IPR012287
1.31E-46	60.75%	P:biological_process		no IPS match
8.19E-26	73.31%	P:biological_process		no IPS match
6.45E-127	68.15%	P:protein metabolic proces		IPR003613; IPR011989; G3DSA:3.30.40.
8.66E-84	62.40%	P:response t	EC:6.3.2.19	IPR003613; G3DSA:3.30.40.10 (GENE3D
0	55.55%	F:phosphoinositide binding		IPR003114; IPR013937
1.32E-32	71.45%	F:DNA binding; P:transcrip		IPR003851
2.90E-138	77.15%	F:binding; P:cell cycle; P:bi		IPR001214; IPR001965; IPR019787; G3D
0	73.95%	P:response to biotic stimu		IPR004841; SignalP (SIGNALP)
0	70.35%	F:binding		IPR002885; SignalP (SIGNALP)

1.03E-45	74.85%	P:multicellular organismal		IPR003340; G3DSA:2.40.330.10 (GENE3
				no IPS match
1.72E-98	70.85%	F:hydrolase activity; C:cytc		IPR008758
1.66E-156	73.55%	F:binding		IPR002885
7.45E-20	65.15%	C:chloroplast; P:biological_		SignalP (SIGNALP)
1.11E-33	56.75%	F:nucleic acid binding; C:m		IPR001878
2.57E-65	94.25%	P:protein mo	EC:6.3.2.19	IPR000608; IPR016135
4.91E-159	60.85%	F:nuclease activity; P:nucle		IPR002885
1.25E-95	72.10%	F:hydrolase activity; F:tran		IPR001140; IPR003439; IPR003593; G3D
0	82.50%	C:membrane; C:Golgi appa		IPR002035; IPR006895; IPR006896; IPR(
3.41E-48	95.65%	C:nucleolus; F:nucleic acid		IPR001163; IPR006649; G3DSA:2.30.30.
4.56E-158	53.60%	C:mitochondrion		IPR007656; SignalP (SIGNALP)
3.99E-69	73.85%	P:cell redox homeostasis;		IPR002109; IPR012335
1.14E-79	71.20%	F:hydrolase a	EC:3.6.3.8; E(	IPR001148; IPR012335; SignalP (SIGNAI
2.85E-40	67.00%	F:catalytic ac	EC:1.11.1.7	no IPS match
4.36E-88	67.95%	P:proteolysis; C:integral to		IPR001563; IPR004326; G3DSA:3.40.50.
1.05E-34	57.10%	F:RNA binding		IPR007930; IPR008395; IPR014002
2.52E-61	89.80%	C:intracellular; F:DNA binc		IPR000164; IPR007125; IPR009072
				no IPS match
2.22E-29	60.00%	F:transferase	EC:2.7.7.49; I	IPR005162
3.84E-101	70.75%	F:transferase	EC:2.7.7.49; I	IPR001584; G3DSA:3.30.420.10 (GENE3
7.24E-16	70.00%	F:nucleotide binding		no IPS match
4.72E-96	97.10%	P:response to	EC:1.11.1.15	IPR000866; IPR012335; IPR019479
6.80E-85	51.15%	F:binding		IPR001841; IPR001965; IPR019787; G3D
2.00E-74	71.75%	C:mitochondrion		IPR018612
2.41E-124	64.55%	C:mitochondrion; P:protein		G3DSA:3.40.50.300 (GENE3D)
3.01E-59	45.00%	F:nucleic acid binding; C:m		IPR004859

D), SignalP (SIGNALP)

2327 (PFAM)

SA:3.10.20.90 (GENE3D)
alP (SIGNALP)

10 (GENE3D)
)

SA:3.30.40.10 (GENE3D)

D)

)SA:3.40.50.300 (GENE3D)
)06900; G3DSA:3.40.50.410 (GENE3D)
100 (GENE3D)

.P)

1820 (GENE3D)

D)

)SA:3.30.40.10 (GENE3D)
