# Supplementary material for: Deletion of beta‐fructofuranosidase (invertase) genes is associated with sucrose content in Date Palm fruit
Source: Plant Direct. 2020 May 27;4(5):e00214. doi: 10.1002/pld3.214 (PMC7251787; doi:10.1002/pld3.214)
Supplement: Supplementary file 4 — Table S4 [file PLD3-4-e00214-s004.pdf]

| INDV1 | INDV2 | N_AaAa | N_AAaa | N1_Aa |
|---|---|---|---|---|
| 109-DGNH-DZ | 117-DGNR-DZ | 906011 | 4002 | 1.07E+06 |
| 109-DGNH-DZ | 46-NBTL-SA | 552888 | 136194 | 1.07E+06 |
| 109-DGNH-DZ | 49-SKR-QA | 530139 | 149938 | 1.07E+06 |
| 109-DGNH-DZ | 64-DGTNR-TN | 941784 | 2212 | 1.07E+06 |
| 109-DGNH-DZ | 70-SKR-SA | 541806 | 141387 | 1.07E+06 |
| 117-DGNR-DZ | 109-DGNH-DZ | 906011 | 4002 | 1.05E+06 |
| 117-DGNR-DZ | 46-NBTL-SA | 531567 | 134810 | 1.05E+06 |
| 117-DGNR-DZ | 49-SKR-QA | 509261 | 148139 | 1.05E+06 |
| 117-DGNR-DZ | 64-DGTNR-TN | 941937 | 3400 | 1.05E+06 |
| 117-DGNR-DZ | 70-SKR-SA | 551078 | 139229 | 1.05E+06 |
| 46-NBTL-SA | 109-DGNH-DZ | 552888 | 136194 | 920829 |
| 46-NBTL-SA | 117-DGNR-DZ | 531567 | 134810 | 920829 |
| 46-NBTL-SA | 49-SKR-QA | 608441 | 28237 | 920829 |
| 46-NBTL-SA | 49-SKR-QA | 608441 | 28237 | 920829 |
| 46-NBTL-SA | 64-DGTNR-TN | 536380 | 143119 | 920829 |
| 46-NBTL-SA | 70-SKR-SA | 559161 | 67591 | 920829 |
| 49-SKR-QA | 109-DGNH-DZ | 530139 | 149938 | 862004 |
| 49-SKR-QA | 117-DGNR-DZ | 509261 | 148139 | 862004 |
| 49-SKR-QA | 46-NBTL-SA | 608441 | 28237 | 862004 |
| 49-SKR-QA | 64-DGTNR-TN | 513250 | 157159 | 862004 |
| 49-SKR-QA | 70-SKR-SA | 538130 | 73454 | 862004 |
| 64-DGTNR-TN | 109-DGNH-DZ | 941784 | 2212 | 1.09E+06 |
| 64-DGTNR-TN | 117-DGNR-DZ | 941937 | 3400 | 1.09E+06 |
| 64-DGTNR-TN | 46-NBTL-SA | 536380 | 143119 | 1.09E+06 |
| 64-DGTNR-TN | 49-SKR-QA | 513250 | 157159 | 1.09E+06 |
| 64-DGTNR-TN | 70-SKR-SA | 566014 | 148247 | 1.09E+06 |
| 70-SKR-SA | 109-DGNH-DZ | 541806 | 141387 | 921793 |
| 70-SKR-SA | 117-DGNR-DZ | 551078 | 139229 | 921793 |
| 70-SKR-SA | 46-NBTL-SA | 559161 | 67591 | 921793 |
| 70-SKR-SA | 49-SKR-QA | 538130 | 73454 | 921793 |
| 70-SKR-SA | 64-DGTNR-TN | 566014 | 148247 | 921793 |

| N2_Aa | RELATEDNESS_PHI |
| --- | --- |
| 1.05E+06 | 0.423724 |
| 920829 | 0.140941 |
| 862004 | 0.119222 |
| 1.09E+06 | 0.43484 |
| 921793 | 0.130091 |
| 1.07E+06 | 0.423724 |
| 920829 | 0.132916 |
| 862004 | 0.111396 |
| 1.09E+06 | 0.437753 |
| 921793 | 0.138264 |
| 1.07E+06 | 0.140941 |
| 1.05E+06 | 0.132916 |
| 862004 | 0.309601 |
| 862004 | 0.309601 |
| 1.09E+06 | 0.124629 |
| 921793 | 0.230095 |
| 1.07E+06 | 0.119222 |
| 1.05E+06 | 0.111396 |
| 920829 | 0.309601 |
| 1.09E+06 | 0.102107 |
| 921793 | 0.21932 |
| 1.07E+06 | 0.43484 |
| 1.05E+06 | 0.437753 |
| 920829 | 0.124629 |
| 862004 | 0.102107 |
| 921793 | 0.134219 |
| 1.07E+06 | 0.130091 |
| 1.05E+06 | 0.138264 |
| 920829 | 0.230095 |
| 862004 | 0.21932 |
| 1.09E+06 | 0.134219 |
