# Supplementary material for: Deletion of beta‐fructofuranosidase (invertase) genes is associated with sucrose content in Date Palm fruit
Source: Plant Direct. 2020 May 27;4(5):e00214. doi: 10.1002/pld3.214 (PMC7251787; doi:10.1002/pld3.214)
Supplement: Supplementary file 5 — Supplementary Material [file PLD3-4-e00214-s005.docx]

**Response to reviewer comments for** “Deletion of beta-fructofuranosidase (invertase) genes is associated with sucrose content in Date Palm fruit  (Malek *et al.)*”

Reviewer #1: 

Summary: The authors used previously collected data - genotyping and sucrose content analysis of 121 date cultivars - to identify factors responsible for altering sucrose content across the date cultivars. They identified a region containing three invertase genes. SNP analysis revealed that two of the three invertase genes were deleted in high sucrose cultivars. Further sequence and sucrose analysis reveals several instances of cultivars that are heterozygous for the deletion - these cultivars' sucrose content is similar to the homozygous non-deletion cultivars (lower sucrose). 

General comments: 
The text is relatively easy to understand and well-written. The story makes sense, and can facilitate future analysis. However, the figures are another matter. Text on several of the figures should be larger, and is not uniform (some serif, some san serif). The labeling is uneven and there are multiple mistakes that should be cleaned up. More attention needs to be paid to the figure legends. Axes and headings of tables need to be better labeled. Species names should be italicized.

We have attempted to standardize the figure fonts and ensure that they are correct/easier to read and don’t crowd. This included adding more text to some of the figure captions to better explain them.

Major points 
Authors mention that there are other genes that could potentially play a role in sucrose content. Are other glycosyl hydrolases that were deleted or a statement to leave some uncertainty?

We have attempted to stress the association here is most likely with only the extreme sucrose content and that even in this case other factors (and invertases) may also play a role. The strongest evidence for this being the presence of medium sucrose content dates that have no deletion of the identified invertases.

It would be interesting, but not necessary to see which of the two deleted invertases were expressed in date fruits. Perhaps existing publically available RNAseq data could be used to analyze gene expression of these two invertases. 

We have added a “Note added in proof” section do the discussion that refers to the recently published manuscript by Hazzouri *et al.* that identified the same deletion as associated with sucrose content in dates. They carried out RNA-seq data specifically on those gene and found high expression at various times of fruit development. In their cultivar, one of the deleted invertases is a pseudo-gene but that is not the case in all cultivars. This is important as it likely contributes to the added variance in sucrose content beyond the extreme phenotype we discuss.

Minor points 
Page 3: "...which are necessary to convert reducing sugars to sucrose(Hasegawa and Smolensky 1970)." I think the sentence has sucrose and reducing sugars flipped. Sucrose is converted to reducing sugars through the action of invertases.

Thank you for catching this. Indeed, the sucrose and reducing sugars were switched and have been corrected.

Data archiving: Manuscript should be updated to include Genbank entries.

Sequences have been deposited in Genbank and have been assigned as (Deglet Noor invertase region sequence) MT009343 and (Khalas invertase region sequence) MT009344. As of 3 Feb, 2020 the sequences are not yet publicly accessible but are in the queue for immediate release.

References need to be edited carefully, species names italicized. 
 Species names have been italicized in the references

Figures: 
o Figure 1 
 o Figure 1 uses different fonts for labeling parts of the figure (A, B, and C are uniform, while D is clearly serif)

The ”d” in Figure 1 has been adjusted to Arial to match the remaining panel lables

o Figure 2 
 o The Y-axis for figure 2 is labeled 'Sucrose (normalized)', but more detail (e.g. units) should be added

We have updated the axis to include the fact that the units are Bradford normalized ion counts
 o The Y-axis should also be expanded, such that the maximum point for the 'Del' samples falls within labeled bounds

The Y-axis has been extended to ensure the maximum points fall within the labeled range.
o Figure legend should include information on the statistical test used to derive the p-value for the level of sucrose comparisons, and should be briefly discussed in the methods

We have added to the figure legend and the text the fact that the statistical test was conducted with a one-sided Wilcoxon rank sum test.

o Figure 3 
 o Axes font is serif, whereas previous figures used sans serif

Fonts in figure 3 were changed to Arial to match the fonts used in other figures.
o Axes labels (PDK... and PDD...) should be explained in the legend

We have changed the axes labels to cultivar names and given the accession numbers for the sequences in the legend

o Figure 4 
 o A/A, A/T, T/T should be explained in the figure legend 
 We have expanded the legend to better explain the genotypes in the figure and their relationship as a tag SNP for the deletion.

o Table 1 
 o Expand on the label for '%' column

We have added the label (% of samples) to indicate the percentage of samples with each genotypes in the two main date palm subpopulations (East and West)
 o The table legend is a bit confusing - I am not sure if there is a mistake. What is the difference between heterozygous for the reference allele and heterozygous for the deletion? Also nomenclature seems to conflict with that used in Figure 2. Need to be consistent in the labeling

Thank you for catching this. Indeed the Hmz label now reads as “homozygous for the reference allele”.
 o Demarcating the columns would help the reader

Columns are now demarcated

o Table 2 
 oTable headings would be helpful

Column headings have been added
 What is the third and fourth column?

The third and fourth columns have been labeled as reference allele and alternative (sucrose containing genotypes) allele
 o Using lines to separate the columns would be useful to the reader 
 Column separations have been added


Reviewer #2: 

This manuscript is written well and describes interesting results on date sugar level in 121 date palm varieties, obtained from different countries and regions. Deglect Noor, a popular variety, has highest level of sugar as compared to other varieties. Sugar level enhances in dry dates as compared to wet types. The question is how sugar level will be affected under the harsh climatic conditions? Any change in temperature during fruit ripening period, how sugar level would be affected

We believe that by using multiple cultivars the affect of variations can be accounted for. At the same time the affect of more dramatic changes in temperature would be beyond the scope of this study.

In vitro propagated date plants, what is the probability influencing sugar level in fruits 
 Certainly somaclonal variation could have an affect on sucrose content in the various dates. Most of the cultivars studied here are from offshoot or tissue culture propagation – both effectively clonal. Because the region identified is a deletion with recessive phenotype we do not expect that there would be an easy path to regain of function. A heterozygote, on the other hand, might lose invertase function through somacolonal variation in methylation resulting in altered sugar patterns.

---------------------------------------------------------------------------- Editor comments: 
As indicated by Reviewer #1, expression-level analysis based on RNA-seq or qPCR is not needed, however, if data is available, please include those findings if possible.

We have added reference and discuss the latest data that shows gene expression of these invertases in date fruit.
